# Morphological Analysis of Reticuloendothelial System in Capuchin Monkeys (*Sapajus* spp.) after Meso-2,3-Dimercaptosuccinic Acid (DMSA) Coated Magnetic Nanoparticles Administration

**DOI:** 10.1371/journal.pone.0140233

**Published:** 2015-11-11

**Authors:** Shélida Vasconcelos Braz, Victoria Monge-Fuentes, Jaqueline Rodrigues da Silva, Carlos Tomaz, Maria Clotilde Tavares, Monica Pereira Garcia, Sônia Nair Báo, Silene Paulino Lozzi, Ricardo Bentes de Azevedo

**Affiliations:** 1 Laboratory of Nanobiotechnology, Department of Genetics and Morphology, Institute of Biological Sciences, University of Brasília, 70910–900 Brasília-DF, Brazil; 2 Department of Genetics and Morphology, Institute of Biological Sciences, University of Brasília, 70910–900 Brasília-DF, Brazil; 3 Primate Center and Laboratory of Neurosciences and Behavior, Department of Physiological Sciences, Institute of Biology, University of Brasília, 70910–900 Brasília, DF, Brazil; 4 Laboratory of Electron Microscopy, Department of Cell Biology, University of Brasília, 70919–970 -Brasília, DF, Brazil; 5 Neurocience Graduate Program, University CEUMA, São Luis, MA, Brazil; RMIT University, AUSTRALIA

## Abstract

Magnetic nanoparticles can be used for numerous *in vitro* and *in vivo* applications. However, since uptake by the reticuloendothelial system represents an obstacle for the achievement of nanoparticle diagnostic and therapeutic goals, the aim of the present study was to evaluate the uptake of dimercaptosuccinic acid coated magnetic nanoparticles by reticuloendothelial system phagocytic cells present in lymph nodes, spleen, and liver tissue and how the presence of these particles could have an impact on the morphology of these organs in capuchin monkeys (*Sapajus* spp.). Animals were intravenously injected with dimercaptosuccinic acid coated magnetic nanoparticles and euthanized 12 hours and 90 days post-injection. Organs were processed by transmission electron microscopy and histological techniques. Samples of spleen and lymph nodes showed no morphological changes. Nevertheless, liver samples collected 90 days post-administration showed slight morphological alteration in space of Disse. Moreover, morphometrical analysis of hepatic mitochondria was performed, suggesting a clear positive correlation between mitochondrial area and dimercaptosuccinic acid coated magnetic nanoparticles administration time. The present results are directly relevant to current safety considerations in clinical diagnostic and therapeutic uses of magnetic nanoparticles.

## Introduction

Magnetic nanoparticles (MNPs) can be used for numerous *in vitro* and *in vivo* applications, such as targeted drug delivery [1–4], magnetic resonance imaging (MRI) [5–7], cell sorting [8, 9], and hyperthermia [10–12]. All of these biomedical applications require that the nanoparticles present high magnetization values, a narrow particle size distribution, and proper surface coating, which must be non-toxic and biocompatible and also allow for a targetable delivery [13]. Although various physical and chemical properties may influence the pharmacokinetics and cellular distribution of MNPs, proteins adsorbed on the surface of the nanoparticle promote its opsonization, leading to aggregation and rapid clearance from the bloodstream [14, 15]. The resultant uptake is due to phagocytosis by the reticuloendothelial system (RES) of the liver, spleen, lymph nodes, and bone marrow [16–19]. Typically, the majority of opsonized particles are cleared in a few minutes by a receptor-mediated mechanism or they are excreted [14]. Thus, the uptake of nanoparticles (NPs) by the RES represents a considerable obstacle for the achievement of MNP diagnostic and therapeutic goals [15].

Previous studies have shown biocompatibility and non-toxicity of magnetic fluids (MF) containing maghemite (gamma-Fe_2_O_3_) core magnetic nanoparticles coated with DMSA (meso-2,3-dimercaptosuccinic acid) (DMSA-MNPs) *in vitro* [20–23] and *in vivo* [24–27]. DMSA was chosen as coating agent due to several reasons: 1) acts as a heavy metal chelant forming strong complexes with the surface layer of the nanoparticles [28, 29]; 2) easy elimination by the urinary system [30]; and 3) its free –SH chemical group is able to bound to several biomolecules, increasing MNP and cell interaction [31]. In addition, DMSA is proven to be a smaller complex when compared with dextran, facilitating its stability *in vivo*, its diffusion, and circulatory processes, decreasing chances of being uptaken by RES phagocytic cells [32].

Considering the potential future use of maghemite core DMSA-MNPs for biomedical applications in humans, a nonhuman primate experimental model was selected to carry out this study. As stated by Monge-Fuentes and collaborators [27], nonhuman primates are relevant preclinical models for human diseases and transplants as they offer an excellent intermediate screen due to their high level of genetic homology, which underlies physiological, biochemical, and anatomic similarities with humans in comparison with any other animal model. However, the drawback related to the use of nonhuman primates as an experimental model is the fact that it is generally associated with a small number of experimental individuals, since we are dealing with a noble animal of difficult and controlled access.

The aim of the present study was to better understand the interaction of maghemite core DMSA-MNPs with the RES phagocytic cells present in the lymph nodes, spleen, and liver tissue of a capuchin monkey experimental model. Also, in view of the accumulation of iron oxide MNPs in these organs, possible morphological alterations were investigated. Methodology used permitted us to achieve the proposed objectives of this work.

## Material and Methods

### Preparation of magnetic nanoparticles

MF containing maghemite (gamma-Fe_2_O_3_) core nanoparticles surface coated with DMSA were utilized. Nanoparticles were synthesized by mixing ferric and ferrous chloride aqueous solutions (2:1 molar ratio) with concentrated ammonia aqueous solution under vigorous stirring. Five mL of DMSA aqueous solution (0.3 M) were added to 25 mL of magnetic suspension in a molar ratio DMSA/Fe = 0.11. NaCl was added to the suspension to reach final salinity concentration of 0.9% w/v. The pH was adjusted to 7.2.

### Ethics statement

All procedures involving animals were conducted according to guidelines from the Brazilian Society of Animal Experimentation (COBEA) and the Principles of Laboratory Animal Care (NIH publication no. 85–23, revised 1996). The authors state that animal care, housing, experimental procedures, and the present study described here were all approved by the Animal Ethics Committee from the Institute of Biology, University of Brasília. All experiments were conducted at the University of Brasília Primate Research Center, Brazil, a facility authorized by the Brazilian Institute for the Environment and Natural Resources (IBAMA) (protocol IBAMA 1/53/1999/000006-2). Animals were also pair-housed at the Primate Research Center in cages with natural substrate, with rope swings and nest boxes, measuring 3 x 3 x 1.8 m, under natural conditions of light and temperature. Animals were given access to food and water *ad libitum*. New supply of food (fruits, fibers, vegetables, worms, and chow) was offered twice daily, in the early morning and at the end of the afternoon, and water was offered by automatic drinking fountains. Animals were fasted overnight before euthanasia. Animals constantly participated in environmental enrichment tasks, as is customarily done in our Primate Research Center.

During the experimental period, animals were observed daily for any behavioral changes or illnesses. Procedures involving magnetic fluid and saline administration, anesthesia, and euthanasia were properly performed by certified veterinarians from the University of Brasília Veterinary Hospital and all efforts were made to avoid animal suffering.

### Study design and experimental animals

A total of three healthy juvenile capuchin monkeys (*Sapajus* spp), ranging from 16 to 18 months of age, were randomly allocated as subjects for histopathological and ultrastructure analysis (control with *n* = 1 and each experimental condition with *n* = 1). A control animal was euthanized 12 h after saline injection and two other experimental animals (EA) were intravenously injected with DMSA-MNPs and euthanized 12 hours (EA12h) and 90 days (EA90d) following administration.

### Experimental procedures

For DMSA-MF and saline administration, animals were first anesthetized with an intramuscular injection of ketamine and xylazine applied at a dose of 10 and 1 mg/kg of body weight, respectively. DMSA-MF was then injected in a concentration of 0.5 mg Fe/kg of body weight. The total dose injected was calculated based on the above-cited concentration and the weights of the animals, which correspond to 1.98 kg (EA12h) and 1.78 kg (EA90d). Saline solution (0.9%) was used as a control substance for control animal. DMSA-MF and saline solution were administered as a single bolus injection into the femoral vein and all injection volumes were kept constant at 1 mL. Twelve hours and 90 days after MF administration, animals were anesthetized with an intramuscular injection of ketamine and xylazine applied at a dose of 10 and 1 mg/kg of body weight, respectively. Afterwards, they were euthanized by intravenous thiopental overdose administration. Once the death of the animals was confirmed, necropsy of the liver, spleen, and lymph nodes was performed.

### Preparation of tissue samples for Light Microscopy

Study organs were fixed in Davidson’s fixative (proportion of 1:3:2:3:1, of glycerin, ethanol, 37–40% solution of formaldehyde (v/v), distilled water, and glacial acetic acid) at 7°C for 24 hours. Once the organs were fixed, they were dehydrated in series of ascending ethanol concentrations (70–100%), clarified in xylene and embedded in Histosec^®^ (Merck, Germany). Semi-serial sections were cut (5μm each) and stained with hematoxylin and eosin (H&E) (NPs appear stained in brown) for histopathological analysis and with Perls’ Prussian blue which stains Fe(III) in bright blue, for iron oxide MNP localization in the tissues. Sections were mounted on glass slides and covered with cover slips. Slides were visualized and analyzed using a Leica^®^ microscope model DM1000 (Leica Microsystems, Switzerland) and digitally photographed using a Leica^®^ DFC280 camera and Leica^®^ Application Suite Version 2.7.0 (Leica Microsystems, Switzerland).

### Preparation of tissue samples for Transmission Electron Microscopy studies

Spleen, liver, and lymph nodes fragments were rinsed with phosphate buffered saline (PBS) (pH 7.2) and then cut into small sections of about 1 mm^3^. Tissues were fixed in a solution containing 2.5% glutaraldehyde, 5 mM CaCl, and 5% sucrose in 0.1 M sodium cacodylate buffer (pH 7.2) at 4°C overnight. Samples were then post-fixed for 1h in osmium tetroxide. Material was dehydrated in series of ascending acetone concentrations and embedded in Spurr’s resin. Ultrathin sections were stained with uranyl acetate and lead citrate. Finally, material was analyzed using a JEOL^®^ 1011C Transmission Electron Microscope (Jeol, Tokyo, Japan) and digitally photographed using Gatan Digital Micrograph^™^ 3.11.0.

### Hepatocyte Mitochondria Morphometry

Mitochondria morphometry was performed in liver of control and experimental tissue samples. For each treatment, twenty electron micrographs were used and five mitochondria from each micrograph were randomly chosen to measure mitochondrial area. For this analysis, the boundary of each mitochondrion was traced to measure its area using the Digital Image-ProPlus 4.5^™^ software (Media Cybernetics, Inc., Silver Springs, MD). In order to compare the hepatocyte mitochondrial areas obtained from the different treatment groups, data were normalized by transforming to Log_10_(1+value) and subjected to ANOVA and Scheffè’s test using StatView statistical software for Windows (SAS Institute Inc., USA). Differences were considered significant when P < 0.05.

## Results and Discussion

### Magnetic nanoparticles characterization

Aliquots from the same magnetic fluid were used in the present study and also in studies conducted by Valois *et al*. [26] and Monge-Fuentes *et al*. [27], members of the same research group conducting the present study. Both works properly characterized the MNPs used in the present study, showing that the maghemite based DMSA-MNPs used here are spherically shaped particles with a narrow diameter range, present the required size boundaries for biomedical applications (8.1 nm average diameter), show stability when in suspension and exist as small aggregates within the MF.

### Light microscopy of spleen and lymph nodes

**Fig 1:**
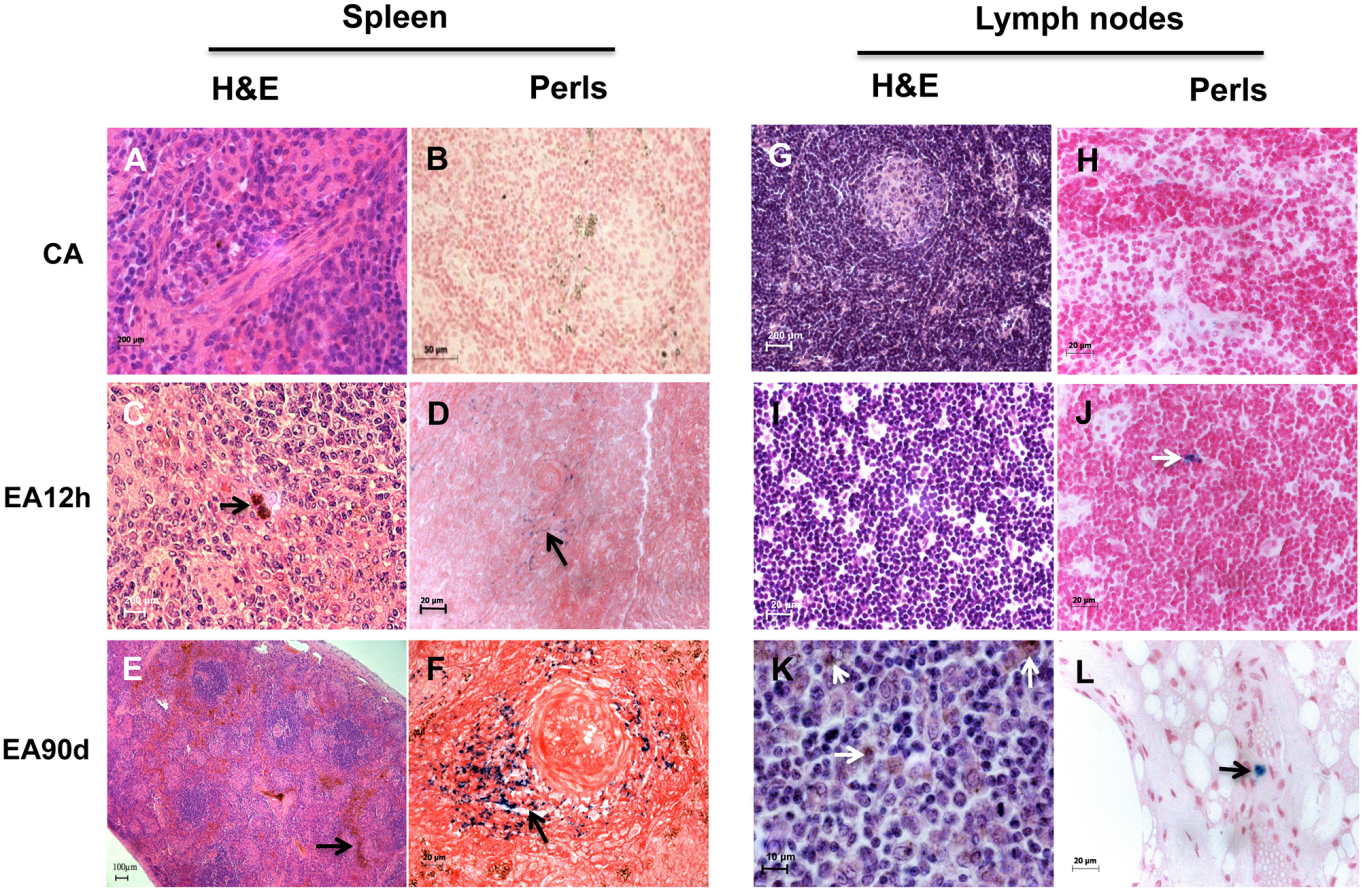
Magnetic nanoparticles coated with meso-2,3-dimercaptosuccinic acid in capuchin monkey reticuloendothelial tissue samples. Tissues were stained with hematoxylin and eosin (H&E) and Perls’ Prussian blue. Spleen and lymph node from control animal (CA) (A, B, G, H,) with typical tissue morphology. Splenic tissue with discrete (C and D) and intense (F) MNPs accumulation in EA12h and EA90d, respectively. Accumulation of hemosiderin (arrow) in (E). Lymph node samples show discrete MNP accumulations (arrows) in the cytoplasm of cells (J, K, L). DMSA: dimercaptosuccinic acid; MNP: magnetic nanoparticle. CA: control animal; EA12h: experimental animal euthanized 12 hours post DMSA-MNP administration; EA90d: experimental animal euthanized 90 days post DMSA-MNP administration. N = 1 animal for control and for each experimental condition. Scale bars: (A, C & G): 200 µm; (B): 50 µm; (D, F, H-J & L): 20 µm; (E): 100 µm; (K): 10 µm.

Light microscopy (LM) for experimental animal euthanized 12 hours post-administration of DMSA-MNP (EA12h) and for experimental animal euthanized 90 days post-injection (EA90d) showed no alterations on spleen and lymph node tissue, showing structure comparable to control animal samples (Fig 1).

Spleen samples from experimental animals EA12h and EA90d presented MNPs in connective tissue near blood vessels, and in both, red and white pulp, being more abundant in EA90d samples (Fig 1C–1F).

Samples from control lymph node presented typical morphology (Fig 1G and 1H), while lymph node experimental samples stained with H&E showed few DMSA-MNP agglomerates in the internal and external cortex (Fig 1I and 1K). When Perls’ Prussian blue method was used to stain the lymph node tissue samples, few DMSA-MNP agglomerates could be visualized in the subcapsular sinus, peripheral cortex, cortical sinus, and capsule (data not shown).

### Electron microscopy of liver

**Fig 2:**
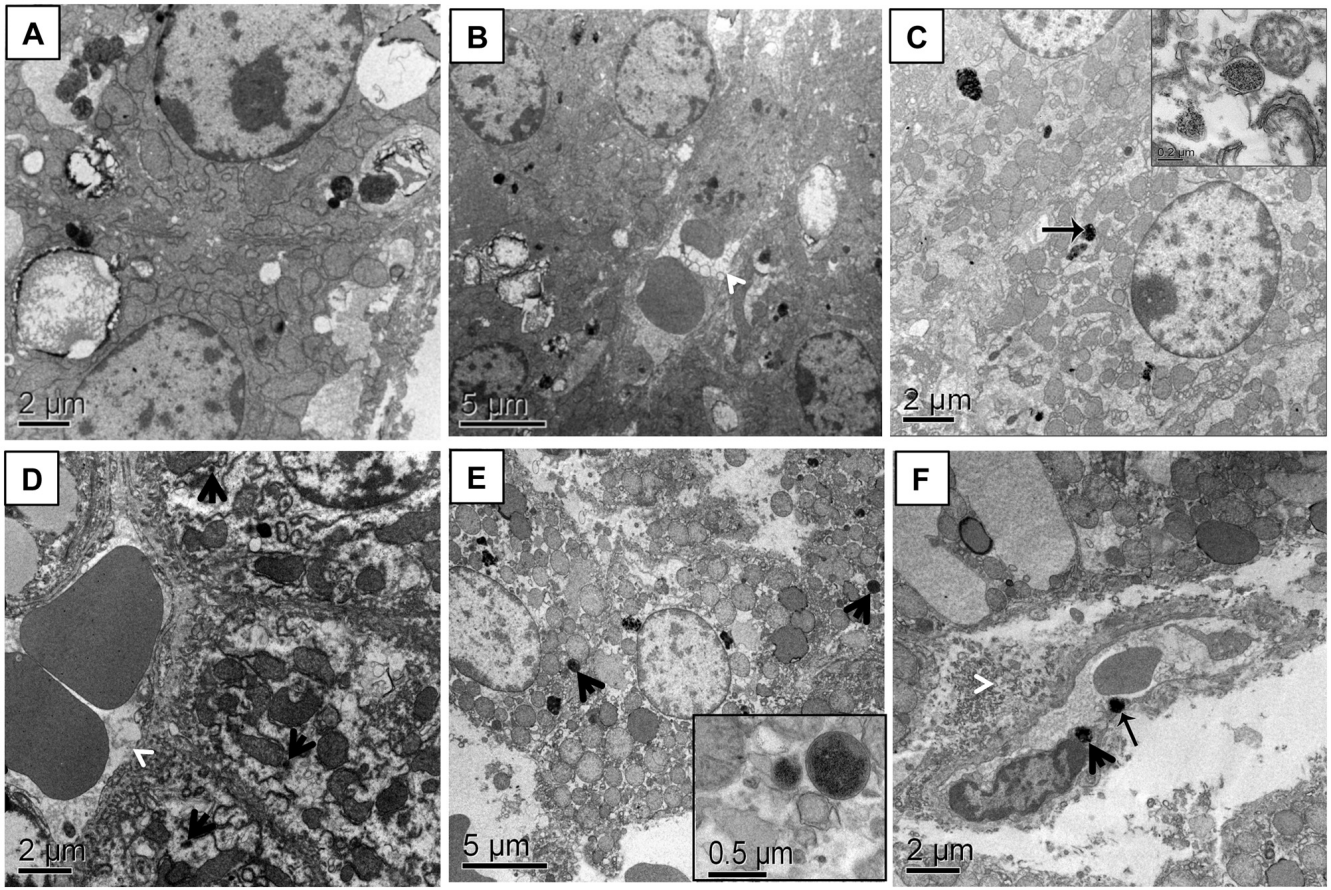
Electron micrograph of capuchin monkey liver tissue. (A and B) Control animal samples with intact hepatocytes. In (B) note the normal appearance of hepatocyte, sinusoids containing red blood cells and normal space of Disse (arrow). (C and D) Liver from EA12h showing the presence of MNPs inside hepatocytes (black arrows), either enveloped by a membrane (inset in C) or free in the cell cytoplasm. In (D) also observe a more dilated space of Disse (white arrowhead). (E and F) Liver from EA90d showing MNPs inside hepatocytes and lysosomes (E). In (F) note the slightly dilated space of Disse (white arrowhead). MNP agglomerates were also observed in the cytoplasm of endothelial cells (black arrows). Scale bars: (A, C, D & F): 2 µm; (B & E): 5 µm. Insets: (C): 0.2 µm and (E): 0.5 µm. N = 1 for control and for each experimental condition.

Ultrastructural analyses from EA12h and EA90d liver samples corroborate LM results seen by Monge-Fuentes and colleagues [27], showing normal and preserved structure in control samples (Fig 2A and 2B) and DMSA-MNP agglomerates in both, endothelial cell cytoplasm and internalized in hepatocytes where MNPs were membrane-bound (Fig 2C and 2D). Our results show MNPs uptaken by Ito cells; however, MNPs were absent in Kuppfer cells. EA90d TEM analysis showed MNPs agglomerates in hepatocyte cytoplasm, internalized or not in lysosomes, and inside endothelial cells (Fig 2E and 2F). Furthermore, in a few hepatocytes from EA90d, cell membrane alterations were visible. This caused the loss of cell characteristic contour, also revealing signs of membrane disintegration (Fig 2E and 2F). As also observed by Monge-Fuentes and collaborators [27], 90 days-post injection of DMSA-MNPs also caused slight augmentation of space of Disse (Fig 2D and 2F). Perhaps, this is due to the fact that sinusoids act as a filter when particles are carried into the liver. This anatomical barrier limits the uptake of large-diameter NPs by parenchymal cells, restricting the passage of smaller sized NPs that are able to leak into the space of Disse from the sinusoidal space through the fenestrations [33]. In spite of the fact that we have no explanation for this increase of Disse space, one possibility is that iron accumulation in the tissue might have caused an increase in blood flow in the sinusoids and thus an overflow of plasma volume to the space of Disse, causing a dilated aspect. However, further studies are necessary to clarify this point.

**Fig 3:**
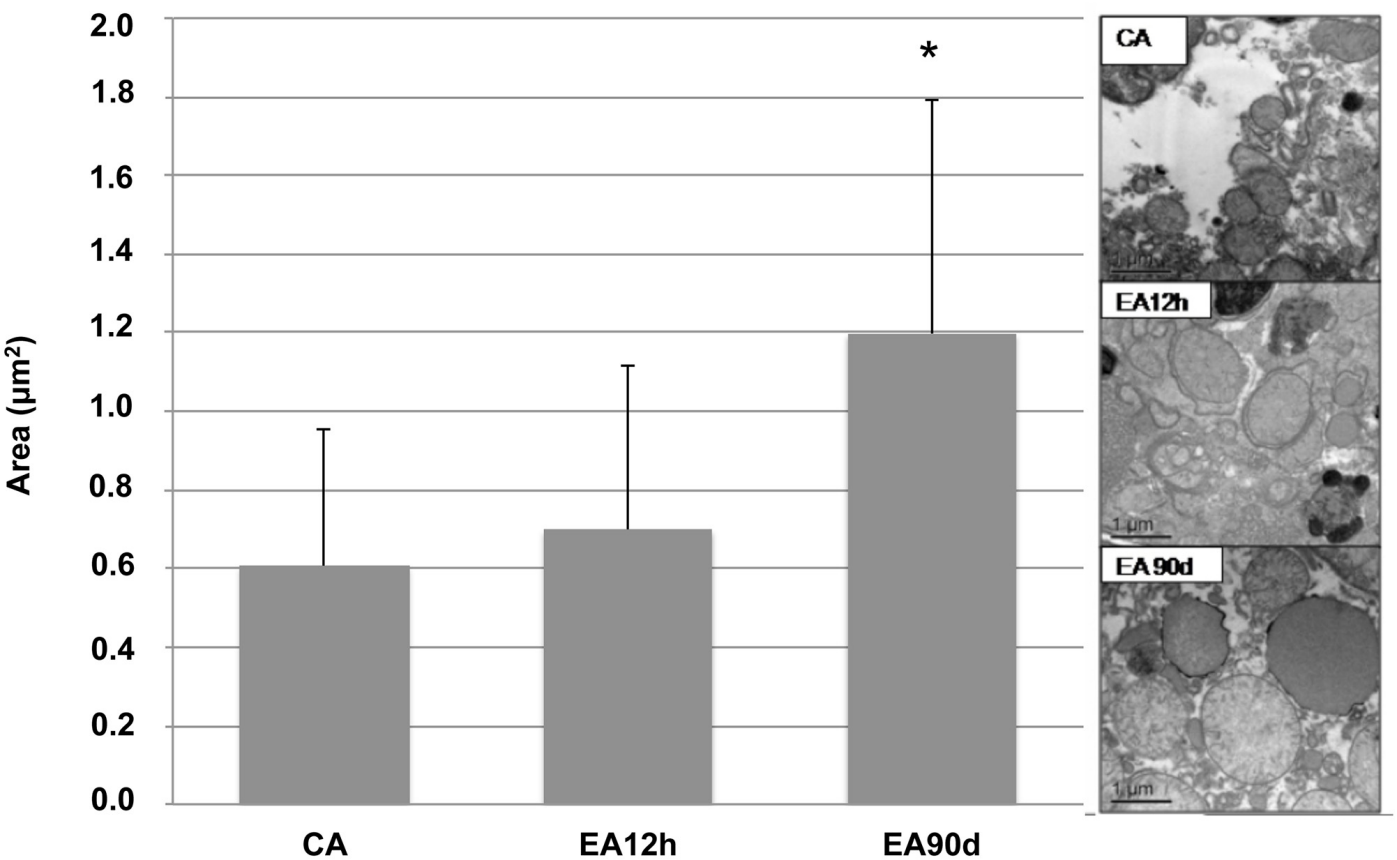
Mitochondrial morphometry of liver samples from control and treated animals. Electron micrographs show hepatocyte mitochondria area (µm^2^) in control animal (CA) (n=1) and experimental animals euthanized 12 hours after magnetic nanoparticle administration (EA12h) (n =1) and 90 days after injection (EA90d) (n =1). Note the swollen appearance of mitochondria in animal EA90d when compared to the other two groups. Graph shows mitochondrial area (mean ± SD) for the different treatments. (*) indicates differences among treatments, P < 0.0001. Scale bars: 1 µm.

DMSA-MNP-treated animals showed hepatocyte mitochondria with a swollen appearance. This lead to a more detailed study in which mitochondrial morphometric analyses were performed (S1 File). Results suggest a positive correlation between mitochondrial area and presence of DMSA-MF in the hepatic tissue. When comparing mitochondrial area for control and EA12h samples, no significant difference was observed. However, when comparing the values obtained for EA90d with control and EA12h samples (Fig 3), significant difference was observed (P < 0.0001).

Peroxidation of mitochondrial membrane provokes an energy production deficit, decreasing ionic pumps action and increasing intracellular calcium accumulation [34–37]. In this way, administration of DMSA-MNPs could have caused an increase in free radicals, as it occurs in iron overloads [38], and consequently, lipid peroxidation related to mitochondrial dysfunction [36, 39]. According to Elloumi and collaborators [40], mitochondrial dysfunctions might be correlated to necrotic-inflammatory lesions in liver with non-alcoholic steatose. However, it is extremely important to note that serum hepatic enzymes levels (bilirrubin, alanine transaminase, gamma-glutamyltransferase, alkaline phosphatase, aspartate transaminase and lactate dehydrogenase) previously studied in Monge-Fuentes and collaborators [27], who worked with the same animals used in the present study, corroborate, along with the morphological aspects presented here, that the mitochondrial alteration observed was not sufficient to cause inflammatory or necrotic lesions.

### Electron microscopy of spleen

**Fig 4:**
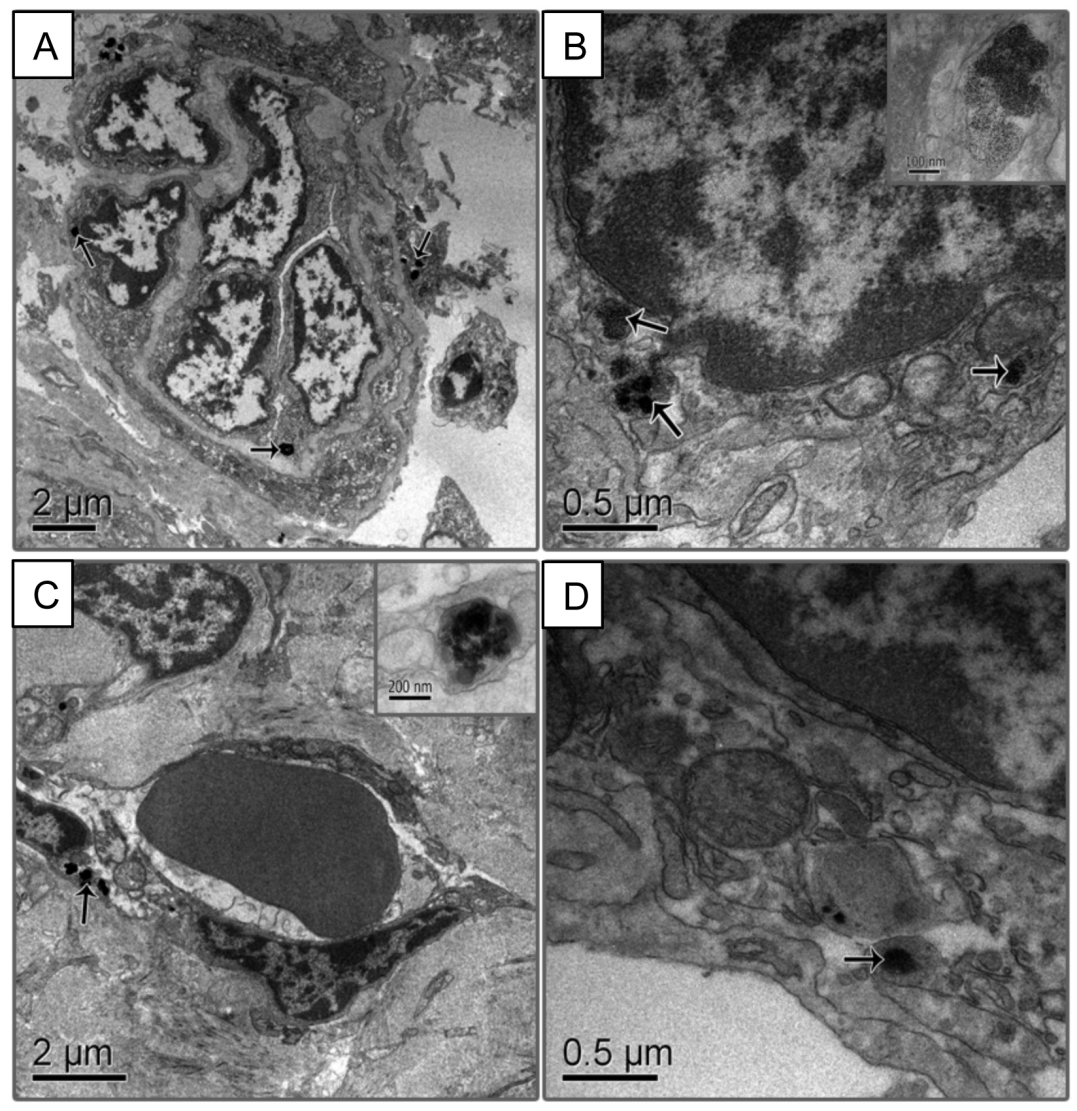
Electron micrographs of capuchin monkey spleen tissue. (A) Control animal showing normal ultrastructure morphology of the spleen and endogenous pigment (arrow). (B and C) EA12h presenting magnetic nanoparticles (arrow) inside a macrophage and endothelial cells, respectively. (D) EA90d with MNPs in macrophage cytoplasm (arrow). Insets show MNPs enveloped by vesicles. Scale bars: (A & C): 2 µm; (B & D): 0.5 µm. Insets: (B): 100 nm and (C): 200 nm.

MNPs were observed enveloped by vesicles. No DMSA-MF was found inside blood vessels. Observation by TEM was consistent with results obtained by LM (Fig 4A–4D).

### Electron microscopy of lymph nodes

**Fig 5:**
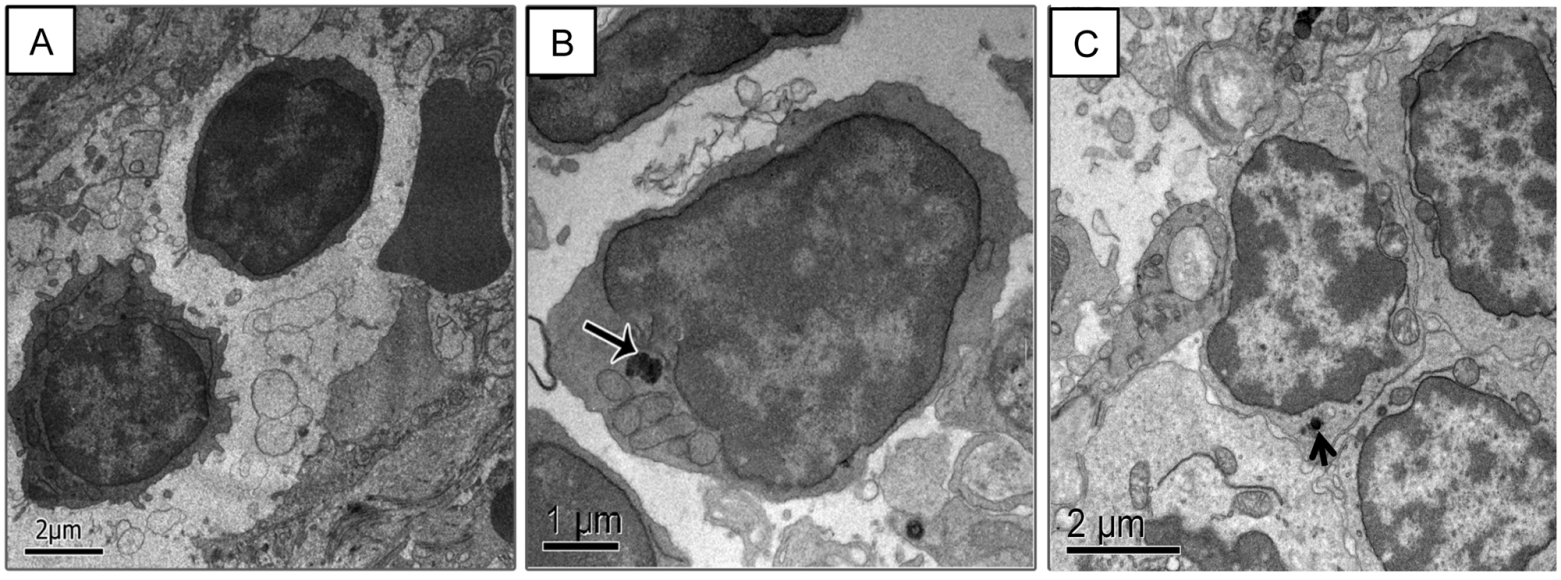
Electron micrograph of capuchin monkey lymph node tissue. (A) Control animal. Lymphocyte showing normal ultrastructure. (B) EA12h with DMSA-MNPs internalized in lymphocyte cytoplasm. (C) EA90d sample, showing few electron dense material internalized in macrophage. Scale bars: (A & C): 2 µm; (B): 1 µm.

Samples from control lymph node presented typical morphology (Fig 5A). MNPs observed in EA12h samples were generally internalized in vesicles (Fig 5B). In lymph nodes of treated animals, DMSA-MNPs were internalized by lymphocytes (Fig 5B) and macrophages (Fig 5C), and were not observed inside blood vessels or endothelial cells. Specifically in the case of EA90d samples, few electron dense material resembling DMSA-MNPs was seen internalized in macrophage and lysosomes (Fig 5C).

### DMSA-MNP interaction with RES phagocytic cells

Studies have shown that foreign particles are opsonized for clearance by RES phagocytic cells located mainly in the liver and the spleen [41]. Therefore, the challenge of using nanoparticles *in vivo* is to bypass this process to allow targeted drug delivery [15, 42, 43]. The major determinants of clearance kinetics and biodistribution of colloidal particles are factors such as surface characteristics, size, and zeta potential [19, 44, 45]. Considering surface characteristics, it is noticeably important to use coatings that reduce opsonization and minimize clearance by RES, leading to improved pharmacokinetic properties. This approach has been used in a variety of nanoparticle systems, generally coated with polymers, in order to improve circulation half-life and enhance drug delivery [41, 46].

Particles with a hydrophobic surface are rapidly opsonized by certain plasma proteins as soon as they are introduced into the bloodstream and are preferentially taken up by the liver, followed by the spleen, and lungs [47]. However, our MNPs were coated with DMSA, which enhances their solubility [48], reducing uptake by macrophages [49–51]. In fact, TEM results showed few DMSA-MNP agglomerates in spleen and lymph nodes macrophages, and no DMSA-MNP agglomerates were observed phagocytosed by Kupffer cells, suggesting that DMSA-MNPs were able to evade the Kupffer cell surveillance.

The fact that MNP agglomerates were observed compartmentalized within the lysosomes of hepatocytes and RES cells in lymph node for EA90d, support the explanation that some MNP agglomerates were probably broken down, with the majority of the iron stored as ferritin and/or hemosiderin, which are antiferromagnetic forms of iron [17, 52–54]. In the same way, Levy *et al*. [43] observed that with superparamagnetic maghemite nanoparticles, the biotransformation of the DMSA-MNP into poorly magnetic iron species over the period of 90 days post-injection represents an advantage when considering DMSA-MNPs as a potential nanomaterial for drug delivery purposes. Levy and colleagues (2011) [43] also hypothesized that the biotransformation of NPs observed takes place in lysosomes and that NPs are processed depending on the availability of iron storage proteins, meaning that NP degradation should be limited by the availability and affinity of iron chelating agents produced intracellularly and the capacity of ferritin or hemosiderin protein to store the released iron. Probably, the remaining superparamagnetic NPs form a reservoir of non-toxic Fe(III) organized in their ferrimagnetic lattice, with poor availability as long as they are not degraded by intracellular iron chelates. On the other hand, the ferritin protein might bind the labile iron species resulting from the dissolution of the nanoparticles and store them into another non-toxic form. Such a mechanism may offer a chance for the cell to regulate the labile iron pool generated by the degradation products of NPs and thus minimize toxicity [55, 56], consistently with the very good tolerance profile of iron oxide NPs.

As mentioned above, particle size also plays an important role in RES activity. The general trend is that smaller particles have a substantially longer lifetime in the blood than larger particles [45]. DMSA-MNPs used in this study presented an average diameter of 8.1 nm (as determined by X-ray powder diffraction) and so, it is expected that this characteristic enables rapid penetration into the cell. However, according to Kulkarin *et al*. [45], particles with less than 200 nm in diameter, the coating material rather than the mean hydrated particle size, may be the major factor determining both, biodistribution and blood half-life of iron oxide particles. Taking into account NP charge, it is well accepted that positively charged nanoparticles have a higher rate of uptake by RES phagocytic cells when compared to neutral or negatively charged formulations [44, 45, 57, 58]. Hence, since DMSA grants a negative charge to the MNP this probably provides a low non-specific internalization rate and long blood half-life.

## Conclusions

Overall, our results propose that the coating, size, and charge of the DMSA-MNPs used in the present study facilitate MNPs ability to reach the organs with reduced uptake by the RES cells. Thus, even though the RES cleared a small portion of the administered DMSA-MNPs, the rest was able to reach the organs. The morphology results presented in this study suggest that DMSA-MNP administered in capuchin monkeys (*Sapajus* spp.) was well tolerated and, therefore, can be considered as a potential and promising nanomaterial platform for future therapeutic and diagnostic use in humans.

## Supporting Information

S1 ARRIVE Checklist (PDF)

S1 File Mitochondria area dataset for control and experimental hepatic tissue samples. (XLS)
